# Comprehensive analysis of scRNA-seq and bulk RNA-seq reveal the characteristics of disulfidptosis and a prognostic signature in BLCA

**DOI:** 10.18632/aging.205686

**Published:** 2024-03-20

**Authors:** Hao Deng, Fan Cheng, Shaoping Cheng

**Affiliations:** 1 Department of Urology, The First People’s Hospital of Jingzhou, Jingzhou 434000, China; 2 Department of Urology, Renmin Hospital of Wuhan University, Wuhan 430060, China

**Keywords:** sc and bulk RNA-seq analysis, risk score signature, disulfidptosis, BLCA

## Abstract

Disulfidptosis is a newly discovered mode of cell death. However, its biological mechanism in bladder cancer (BLCA) is still uncharacterized. In this investigation, we firstly examined the expression and mutation of disulfidptosis-related genes (DRGs) in BLCA. Two disulfidptosis phenotypes associated with DRGs expression patterns and immune cell infiltration were built. A disulfidptosis risk score signature was constructed based on ten differentially expressed genes (DEGs) between the disulfidptosis subtypes, which allowed patients to be stratified into high- and low-risk groups. We further confirmed that the disulfidptosis risk score signature has great power to predict prognosis, immune cell infiltration, and immunotherapy efficacy in BLCA. Additionally, we analyzed the differences in therapeutic sensitivities between high- and low-risk groups concerning targeted inhibitor therapy and immunotherapy. Analysis of single-cell RNA sequencing was conducted of the ten hub DRGs. Of the ten genes, we found that DUSP2 and SLCO1B3 were differentially expressed in BLCA tissues and adjacent normal tissues, and were markedly associated with patients’ prognosis. Functional experiments revealed that overexpression of DUSP2 or knockdown of SLCO1B3 significantly inhibited cell proliferation, migration, and invasion in BLCA cells. In all, we present a fresh disulfidptosis-related prognostic signature, which has a remarkable capacity to characterize the immunological landscape and prognosis of BLCA patients.

## INTRODUCTION

Bladder cancer (BLCA) has high risks of recurrence and mortality [1]. In 2022, the new cases of BLCA are about 92,000 in China [2]. BLCA can be classified into two patterns pathologically. Specifically, 75% of BLCA patients are diagnosed with non-muscle invasive bladder cancer (NMIBC), and the rest are muscle-invasive bladder cancer (MIBC) [3]. Early diagnosis and timely treatment can improve outcomes for BLCA patients. The common treatments for high-risk NMIBC and MIBC include radical cystectomy, cisplatin-based chemotherapy, and immunotherapy [4]. Cisplatin-based chemotherapy can improve the overall survival of advanced BLCA patients [5]. Immunotherapy against PD-1/PD-L1 is also a promising treatment for BLCA [6]. Despite the advancement in therapeutics, the response duration to chemotherapy and immunotherapy is limited [6, 7]. BLCA patients experience unfavorable prognosis even after chemotherapy or immunotherapy. Therefore, it is an urgent need for us to investigate novel biomarkers.

Disulfidptosis is a pioneering approach to cellular demise that uniquely influences apoptosis in cancerous cells through the alteration of cytoskeletal protein structures. Furthermore, this process is intricately linked to changes in the cellular oxidation-reduction (redox) balance. Research highlights a profound correlation between cancer progression and disulfide bond metabolism, whereby elevated levels of SLC7A11 counteract disulfidptosis under glucose-deficient conditions by facilitating cystine uptake [8, 9]. The essence of disulfide metabolism lies in its crucial role in managing intracellular redox activities, which are governed by the dynamics of disulfide bond formation and disruption. Oxidative stress, a common challenge in cancer cells, precipitates complexities in disulfide metabolism, thereby impacting cell survival and multiplication [10, 11]. As such, disulfidptosis represents an innovative strategy for controlling cancer development, yet further research is needed to elucidate its unique mechanisms. The metabolic intricacies of disulfide within oncogenic cells are linked to a spectrum of biological processes, including immune evasion, metastatic spread, and therapy resistance [12, 13]. Additionally, the role of disulfidptosis in eliciting tumor immune responses merits attention. It has the capacity to trigger tumor immune cells, augmenting immune attack by tumor-specific T cells, enhancing both antibody-mediated and cell-mediated immunity, and potentially refining cancer therapies. Identifying new biomarkers associated with disulfidptosis could pave the way for connecting disulfide metabolism-related targets and pathways to cancer vulnerability.

Recent studies have shown that disulfidptosis is closely related to BLCA. The most recent study constructed a convenient prognostic risk model by combining disulfidptosis and M2 tumor-associated macrophages (TAMs) to facilitate individualized treatment and drug choices for BLCA patients [14]. However, no other study has assessed the role of disulfidptosis in BLCA. This study aimed to investigate the potential biological mechanism of disulfidptosis. Specifically, expression and mutation of DRGs in BLCA were investigated. The results showed two distinct disulfidptosis phenotypes to be associated with DRG expression patterns and immune cell infiltration. A novel disulfidptosis risk signature was also developed based on ten DEGs between disulfidptosis subtypes to predict the prognosis of BLCA patients. Additionally, we defined two phenotypes and conducted single-cell RNA sequencing (scRNA-seq). However, only two of the ten hub genes (DUSP2 and SLCO1B3) were differentially expressed at the single-cell level in BLCA tissues and adjacent normal tissues. DUSP2 and SLCO1B3 were also strongly associated with patient prognosis. Finally, functional experiments were performed to explore the effect of DUSP2 and SLCO1B3 on the malignant behaviors of BLCA cells. This study provides new ideas for individualized management of BLCA patients based on disulfidptosis.

## MATERIALS AND METHODS

### BLCA datasets and preprocessing

In this research, a comprehensive collection of 1020 BLCA samples was compiled from TCGA, GSE13507, GSE31684, GSE32894, and GSE37815 datasets. A diverse dataset was assembled, encompassing somatic mutations, copy number variations (CNVs), and detailed survival profiles. The analysis utilized normalized matrix files from the GEO database and TCGA gene expression metrics, calculated as fragments per kilobase million (FPKM) values, which were converted to transcripts per million (TPM) values for unified analysis. This study employed the ComBat algorithm via the R ‘SVA’ package to mitigate batch effects and other nonbiological variations [15]. Single-cell RNA sequencing data from GSE135337 [16] were also integrated, adhering to established quality control and postanalysis protocols. Cell annotations were carried out per the original methodology, with the “FindAllMarkers” and “FindMarkers” functions applied to conduct Wilcoxon tests for cluster-specific gene expression identification. Gene expression visualization was achieved using the ‘featureplot’ function. The scRNA-seq data from GSE130001 are accessible through the Tumor Immune Single-cell Hub (TISCH) database (http://tisch.comp-genomics.org/home/).

### Unsupervised clustering based on DRGs

A diverse set of 26 DRGs was compiled from various studies to explore distinct patterns of disulfidptosis associated with these genes [9]. Using the “ConsensusClusterPlus” package in R, hierarchical agglomerative clustering analysis was applied. This approach utilized stability metrics from unsupervised analysis to ascertain the optimal number of clusters and their members. By performing the analysis across 1,000 repetitions, the reliability of the stability metrics was ensured, confirming the dependability of the clustering results [17].

### Gene set variation analysis

Using the “GSVA” package in R, enrichment analysis was carried out to identify variations in biological processes among disulfidptosis subtypes. This method, which is both nonparametric and unsupervised, allowed for assessment of changes in the activity of pathways and biological processes across different gene expression datasets. The analysis incorporated Gene Ontology (GO) and Kyoto Encyclopedia of Genes and Genomes (KEGG) datasets from the MSigDB database for evaluating disulfidptosis-associated pathways, which were illustrated through heatmap visualizations [18].

### Immune cell infiltration

The “GSVA” package in R was used for single-sample gene-set enrichment analysis (ssGSEA) to quantify immune cell infiltration (ICI) levels within each sample. Initially, data on expression of immune cell markers were obtained from a study by Charoentong. The ssGSEA method was then applied to calculate an enrichment score reflecting the relative abundance of immune cells in each sample. This enabled comparative analysis of ICIs across different disulfidptosis subtypes based on the calculated scores [19].

### Identification of DEGs between disulfidptosis subtypes in BLCA

Differential expression analysis of genes across disulfidptosis subtypes within BLCA was conducted using the “Limma” package in R [20]; the threshold for significance was set at an adjusted *p* value of less than 0.01. Furthermore, to understand the roles of these DEGs, functional annotation was carried out with the “clusterProfiler” package in R, focusing on GO and KEGG analyses [21].

### Construction of the disulfidptosis score

Prognostically significant DEGs were identified through univariate Cox regression analysis to assess their predictive value. Subjects were divided into two gene clusters, A and B, and subsequently split into a training group (*n* = 452) and a test group (*n* = 452). A risk score model for DEGs was formulated using the training group, applying the LASSO technique to minimize overfitting and refine the DEGs to a final list of ten genes for prognostic modeling. The model’s risk score, or disulfidptosis score, was calculated with the following formula: Risk score = Σ (Expi × Coefi), where Coefi represents the coefficient of risk and Expi is the expression level of each gene. This division created low- and high-risk categories for patient stratification. Survival and ROC curve analyses were conducted within the training cohort, with further validation in both the test cohort and the entire cohort. A nomogram, designed using the “RMS” R package, facilitated individual survival probability predictions, as complemented by calibration curves to forecast 1-, 3-, and 5-year survival probabilities for bladder cancer patients.

### Prediction of multiple therapeutic sensitivities

This research sought to examine differences in the response to targeted inhibitor (TI) therapies and immunotherapy between individuals classified into high-risk and low-risk groups. The median inhibitory concentration (IC50) for TIs, such as those in the Notch, Hedgehog (HH), and Wnt pathways, was assessed using the “pRRophetic” R package [22]. Wilcoxon rank-sum tests were applied to compare IC50 values across the risk groups. A comprehensive evaluation of genes linked to the immune response, including genes related to effector cells, MHC complexes, and regulatory immune factors, was undertaken to assess immunogenicity. This assessment was refined through machine learning techniques to ensure accuracy. The immunophenoscore (IPS), an indicator of immunotherapy response, was compared between groups treated with various immunotherapeutic approaches. An exhaustive review of databases on immunotherapy-treated subjects was conducted [23]. Specifically, data from the metastatic urothelial carcinoma study (IMvigor210) were processed with the “IMvigor210CoreBiologies” and “edgeR” R packages, followed by normalization and transformation using “limma’s” voom method. Additionally, prognostic and immunotherapy response data, including disulfidptosis scores from the IMvigor210 cohort, were compiled [24].

### scRNA-seq analysis of hub DRGs

Single-cell RNA sequence analysis was conducted using the “Seurat” and “SingleR” R packages. Quality control was stringently applied to the raw cell matrix, demanding that genes must be present in at least five cells, cells must exhibit more than 100 genes, and cells must be removed i mitochondrial gene expression exceeds 5%. Normalization of the scRNA-seq data was performed using “Seurat,” adopting “LogNormalize” as the normalization technique. This process transformed the data into a Seurat object, with “FindVariableFeatures” pinpointing the top 1,500 variable genes. Principal component analysis (PCA) was then applied to these genes using “RunPCA,” with dimensionality reduction focusing on these key genes. The JackStraw method was used to determine significant components, and the first 15 PCs were selected for clustering analysis. “FindNeighbors” and “FindClusters” were pivotal in the clustering, leveraging Euclidean distances within PCA for graph construction and neighbor identification. The “RunTSNE” function facilitated t-SNE for improved clustering visualization. Differential expression across clusters was assessed with the Wilcoxon-Mann-Whitney test via “FindAllMarkers,” with stringent criteria for marker identification.

### Cell culture and transfection

Three human BLCA cell lines (UMUC-3, 5637, and T24) and a normal human uroepithelial cell line (SV-HUC-1) were acquired from the Chinese Academy of Sciences. The cells were cultured in RPMI-1640 medium supplemented with 10% FBS and 1% penicillin/streptomycin at 37°C and 5% CO_2_. Overexpression and silencing plasmids were constructed by GenePharma (Shanghai, China). Cell transfection was performed following the manufacturer’s instructions. The transfection efficiency was evaluated via qRT-PCR and western blotting.

### qRT-PCR

Total mRNA was extracted from cells using TRIzol (Bioshape). Reverse transcription was performed using Fasting gDNA Dispelling RT SuperMix Kit with the following experimental conditions: initial temperature of 42°C for 15 minutes and 95°C for 3 minutes. qRT-PCR was performed using a real-time PCR system (Applied Biosystems Life Technologies, USA). The 2^ΔΔCT^ method was used to assess relative expression levels. The following primers were used:

DUSP2 forward primer: 5′-TGGACGAGGCCTTTGACTTC-3′; reverse primer: 5′-GAAGAGCACCAGGTCGGAAA-3′.

GAPDH forward primer: 5′-AGTCCACTGGCGTCTTCAC-3′; reverse 5′-GAGGCATTGCTGATGATCTTGA-3′.

SLCO1B3 forward primer: 5′-CAGCACACTTGGGTGAATGC-3′; reverse primer: 5′-AGCCCAAGTAGACCCTTCCA-3′.

GAPDH forward primer: 5′-AGGAGTAAGACCCCTGGACC-3′; reverse primer: 5′-ACATGGCAACTGTGAGGAGG-3′.

### CCK-8 assay

The effect of DUSP2 and SLCO1B3 on cell proliferation in BLCA was assessed using a CCK-8 assay. Briefly, cells were digested and then seeded into a 96-well plate (1000 cells/well). The cells were treated with CCK-8 (10 μL/well) and incubated at 37°C for 24, 48, or 72 h. Absorbance at 450 nm was assessed using a microplate reader.

### Ethynyl-2′-deoxyuridine (EdU) assay

Cells were seeded and cultured and then incubated with EdU working solution. The cells were fixed with 4% paraformaldehyde for 30 minutes, stained with an EdU kit (Beyotime), and visualized under a fluorescence microscope (Olympus).

### Wound-healing assay

Cells (1 × 10^6^ cells) were seeded in a 6-well plate at 5% CO_2_ and 37°C until they reached 90% confluence. The cells were incubated and then scraped with a pipette tip to create wounds. The gap distance was measured. A microscope was used to acquire images (Olympus, Japan).

### Transwell invasion assay

The invasion ability of BLCA cells was assessed using Transwell chambers (Corning Life Sciences). Briefly, the cells were seeded into the upper chamber coated with Matrigel (BD Biosciences) to form a matrix barrier. The Transwell membrane was then fixed with 4% paraformaldehyde, followed by 0.5% crystal violet staining. The invading cells were counted using ImageJ software.

### Western blotting

First, cells were lysed in buffer (Beyotime). Total protein was extracted, separated by SDS-PAGE and transferred to a PVDF membrane. The membrane was blocked with 5% skim milk and then incubated with primary antibodies against SLCO1B3 (Cat No. 66381-1-Ig, 1:4000), DUSP2 (Cat No: 27327-1-AP, 1:1000) and GAPDH (Cat No. 60004-1-Ig, 1:20000) from Proteintech Company. The membranes were washed and incubated with secondary antibodies (1:1000, Abcam). The protein bands were scanned, and images were obtained.

### Statistical analyses

R software (version 4.0.5) was used for statistical analyses. The R packages “maftool” [25] and “rcircos” [26] were used to identify gene mutations and CNVs, respectively. *P* < 0.05 was considered to indicate a statistically significant difference.

## RESULTS

### Genetic variations in DRGs among BLCA patients

**Figure 1:**
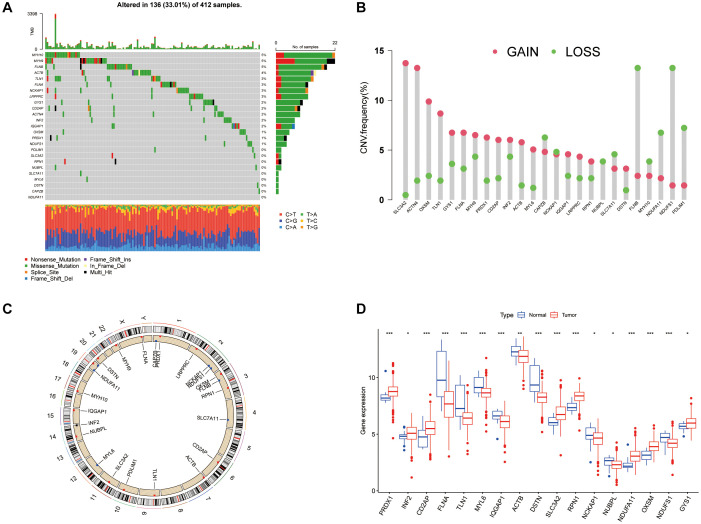
**Genetic and expression variation landscape of DRGs in BLCA.** (**A**) Mutation frequency of DRGs in 412 BLCA patients from the TCGA cohort. (**B**) CNV frequency of DRGs. (**C**) CNV positions of DRGs on chromosomes. (**D**) Expression of 26 DRGs in normal and BLCA tissue. ^*^P < 0.05, ^**^P < 0.01, ^***^P < 0.001.

An examination of 26 DRGs from recent studies revealed mutations in 136 of 412 samples analyzed. Among these genes, MYH10 was the most frequently mutated (Figure 1A). In the context of BLCA, CNVs within DRGs were notably prevalent, with a predominant occurrence of deletions and a substantial presence of amplification events in nearly half of the DRGs. Specifically, genes such as SLC3A2, ACTN4, OXSM, and TLN1 showed significant amplification, whereas the remaining genes were more prone to deletions (Figure 1B). Further investigation highlighted the chromosomal distribution of CNVs across the DRGs and explored how genetic alterations influence mRNA expression levels in BLCA, suggesting that CNVs might modulate DRG expression. Amplifications in genes such as SLC3A2, OXSM, and GYS1 were linked to increased expression in cancer tissues, whereas genes such as NDUFS1 and NCKAP1 showed reduced expression, underscoring the diverse impact of DRG expression on BLCA pathology (Figure 1C, 1D).

### Two distinct disulfidptosis processes: phenotypes associated with DRG expression patterns and immune cell infiltration characteristics and biological behaviors

**Figure 2:**
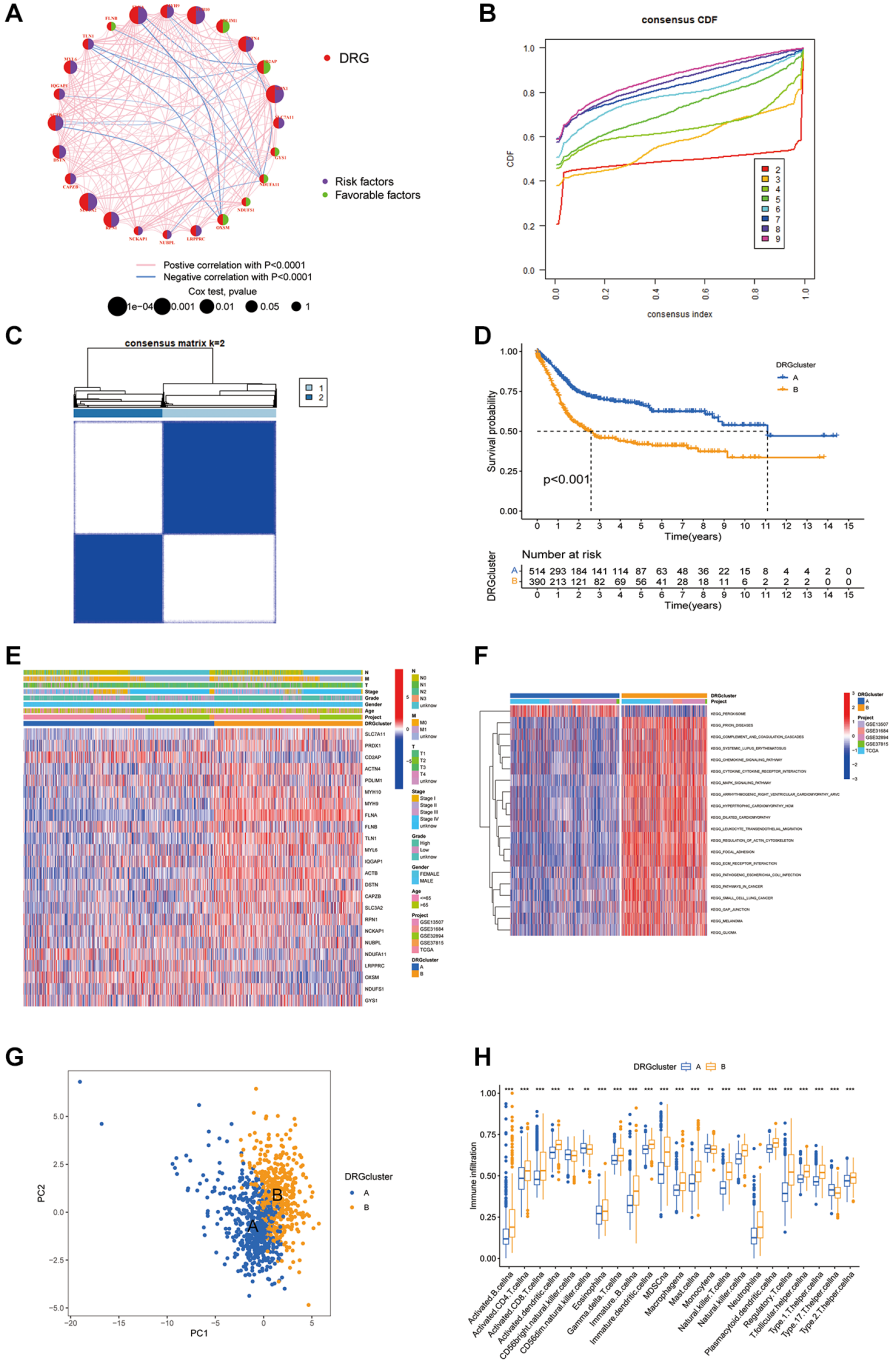
**Patterns of disulfidptosis and biological characteristics of each pattern.** (**A**) Interactions between DRGs. (**B**) Cumulative distribution function curve. (**C**) Consensus matrix of the BLCA cohort with k = 2. (**D**) Kaplan-Meier curves show that the disulfidptosis pattern is significantly associated with OS in 904 patients in the meta-cohort. (**E**) Unsupervised clustering analysis was performed on 26 DRGs. (**F**) GSVA enrichment analysis showed the activation states of biological pathways in distinct disulfidptosis patterns. (**G**) Principal component analysis of the DRG clustering patterns. (**H**) The abundance of each TME infiltrating cell in two disulfidptosis patterns. ^*^P < 0.05, ^**^P < 0.01, ^***^P < 0.001.

Through univariate Cox proportional hazards regression and correlation analyses, intricate connections, relationships, and their prognostic relevance among DRGs were evaluated. The analysis pinpointed 18 genes closely tied to functional outcomes as significant predictors of BLCA patient survival outcomes (*P* < 0.05), with a noted positive correlation among DRGs sharing prognostic similarities. Specifically, a substantial correlation was noted between expression of FLNA and that of several genes, whereas a negative correlation was found between genes associated with positive and negative survival outcomes. In particular, the beneficial effect of OXSM was inversely related to risk factors such as ACTB, TLN1, and FLNA (Figure 2A). Furthermore, distinct expression patterns of DRGs in patients facilitated identification of two unique disulfidptosis clusters, A and B, through “ConsensusClusterPlus” analysis, with Cluster A showing more favorable prognosis (Figure 2B–2D). Heatmaps showcased the variance in DRG expression between these clusters, while KEGG and GSVA analyses highlighted the pronounced stromal signals in Cluster B (Figure 2E, 2F). PCA further underscored the potential of the DRGs to distinguish between BLCA patients and healthy controls. Analysis of ICIs revealed a significant diversity of immune cells in Cluster B, suggesting a complex immunological landscape within the tumor microenvironment indicative of the role of disulfidptosis in driving immunosuppressive and inflammatory responses (Figure 2G, 2H).

### Secondary clustering using DEGs and identification of prognostic-related subtypes

**Figure 3:**
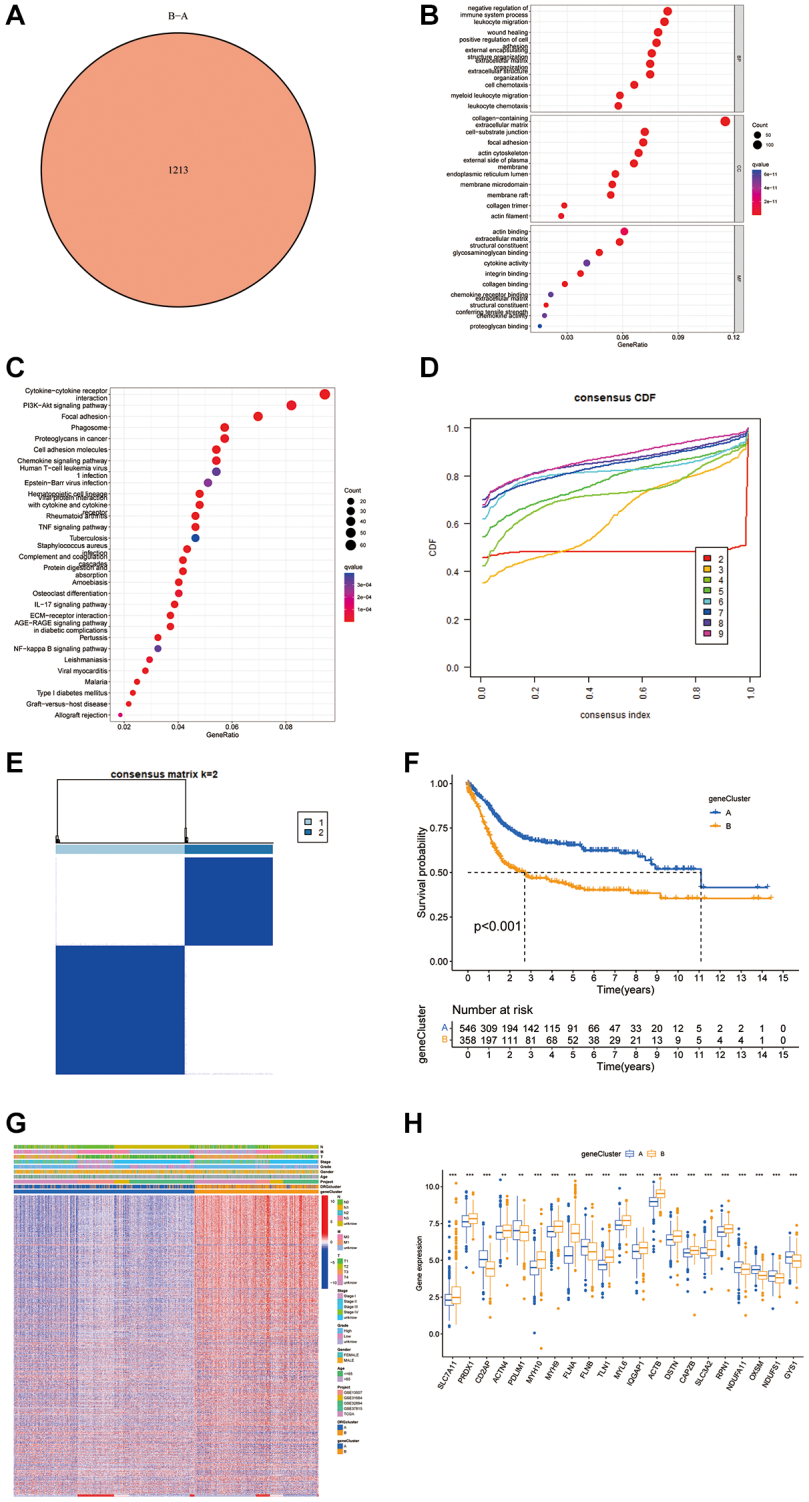
**Analysis of DEGs and functional annotation of disulfidptosis.** (**A**) DEGs between gene groups. (**B**, **C**) Functional annotation of DRG cluster-related DEGs using GO and KEGG enrichment analysis. (**D**) Cumulative distribution function curve. (**E**) Consistency matrix of BLCA sequences with k = 2. (**F**) Kaplan-Meier curves showed that the disulfidptosis genomic phenotype was significantly associated with OS of BLCA patients. (**G**) Unsupervised clustering of DEGs to classify patients into different genomic subtypes. (**H**) Expression of 26 DRGs in gene cluster A and B from the meta cohort. ^*^P < 0.05, ^**^P < 0.01, ^***^P < 0.001.

By utilizing the “Limma” R package, 1213 DEGs were identified, shedding light on the diverse biological activities linked to disulfidptosis phenotypes (Figure 3A). GO and KEGG analyses highlighted the critical involvement of these DEGs in immune-related pathways within the tumor microenvironment (TME) (Figure 3B, 3C). Subsequent univariate Cox analysis of these genes underscored their prognostic significance in BLCA, and 923 DEGs were identified for further prognostic exploration. Through unsupervised clustering of these DEGs, patients were categorized into two groups, revealing notable differences in survival outcomes linked to disulfidptosis-induced variations. This bifurcation revealed a marked survival advantage for patients in one cluster, aligning with specific disulfidptosis patterns; the other cluster indicated poorer prognosis (Figure 3D–3F). The disparity in DRG expression between these clusters validates the influence of disulfidptosis on disease progression (Figure 3G, 3H).

### The disulfidptosis score and its predictive power for prognosis in multiple cohorts

**Figure 4:**
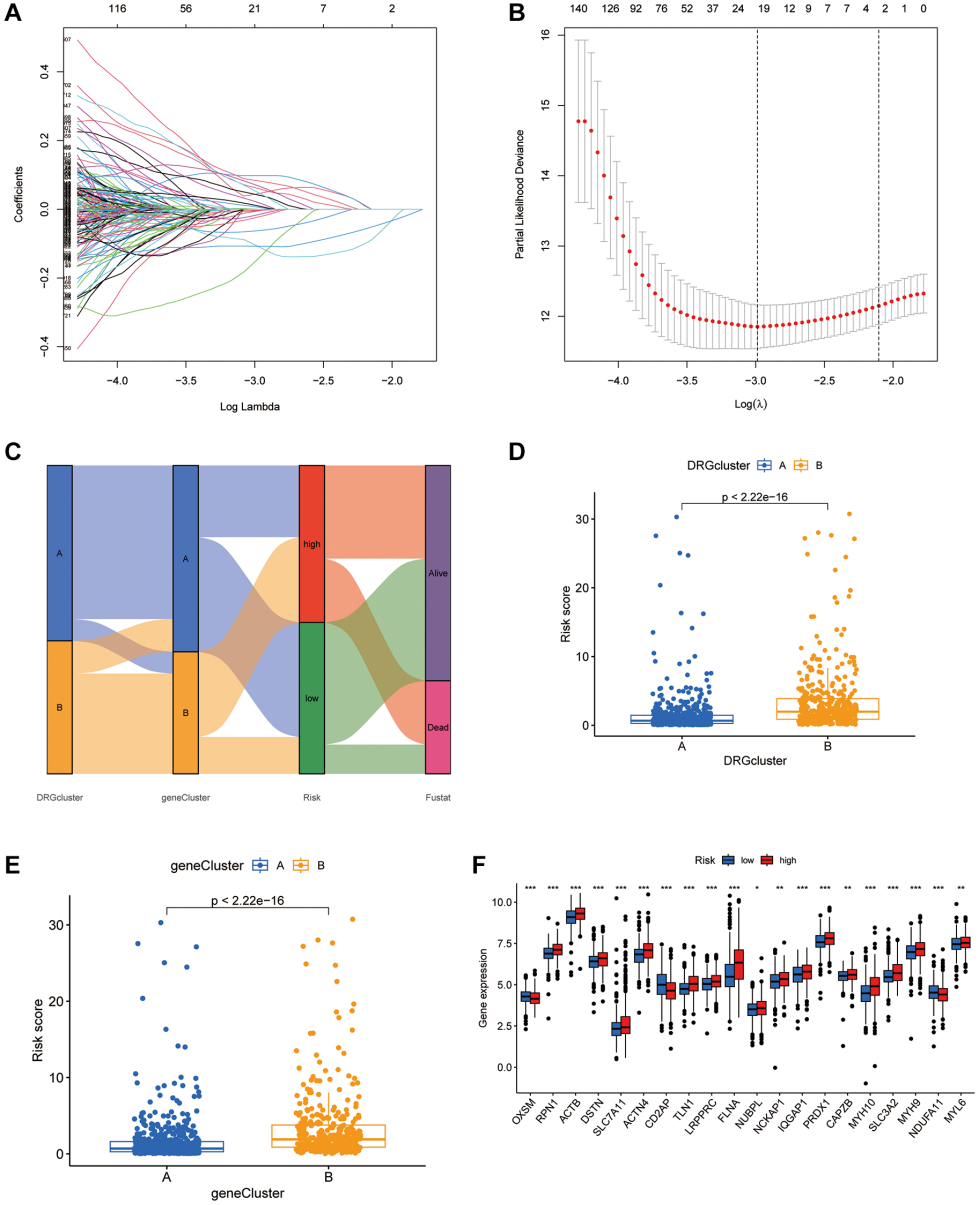
**Establishment of the DRGs risk score and its interaction with tumor clinicopathological characteristics.** (**A**) Cross-validation was performed for tuning parameter selection in the LASSO Cox regression model. (**B**) LASSO coefficient profiles of the DRGs. (**C**) Sankey diagram showing the changes of DRG clusters, survival status, gene cluster, and disulfidptosis score. (**D**) Differences in disulfidptosis score between 2 DRG clusters. *P* < 0.001). (**E**) Differences in disulfidptosis score among 2 gene clusters. The Kruskal-Wallis test was used to compare the statistical difference between 2 gene clusters. (**F**) The difference of DRG expression in low-risk and high-risk group. ^*^P < 0.05, ^**^P < 0.01, ^***^P < 0.001.

**Figure 5:**
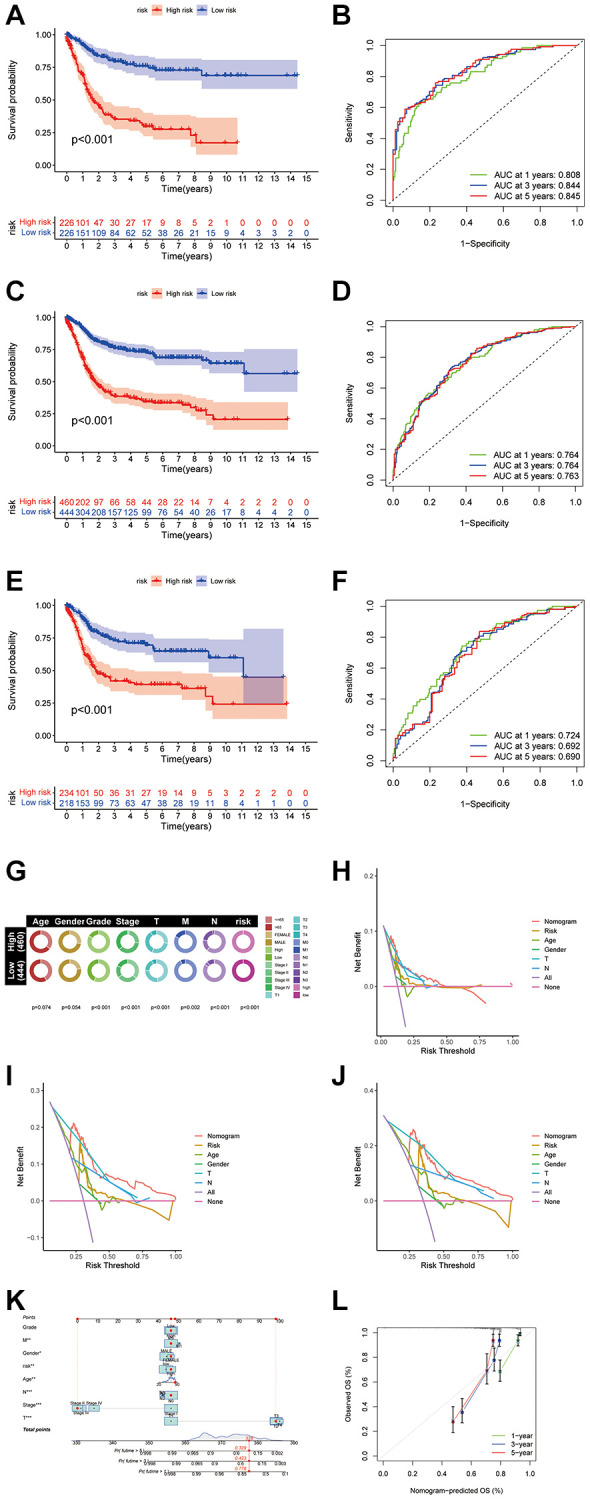
**Construction of the nomogram score system and its clinical predictive performance.** (**A**) Kaplan-Meier curves showed that the disulfidptosis genomic phenotype was significantly associated with OS of patients in the training group. (**B**) ROC curves of the nomogram score system for BLCA patients in the training cohort. (**C**) Kaplan-Meier curves showed that the disulfidptosis genomic phenotype was significantly associated with OS of BLCA patients. (**D**) ROC curves of the nomogram score system for overall patients. (**E**) Kaplan-Meier curves showed that the disulfidptosis genomic phenotype was significantly associated with OS of patients in the validation group. (**F**) ROC curves of the nomogram score system for patients in the testing cohort. (**G**) Pie chart showing the survival status and clinical stage of patients in high and low-risk groups. (**H**–**J**) DCA curves of the nomogram score system for overall patients with 1, 3, 5 years. (**K**) Nomogram score system for overall patients. (**L**) Prediction curves of the nomogram score system for overall patients.

A disulfidptosis score was established to explore disulfidptosis traits thoroughly. Initially, participants were allocated to a training set or a test set. LASSO regression analysis of 923 genes revealed ten genes significantly linked to disulfidptosis outcomes (Figure 4A, 4B). Through multivariate Cox regression, these genes were classified into risk categories, shaping the prognostic landscape. Notably, gene coefficients were meticulously calculated, facilitating a nuanced understanding of their prognostic impact. This led to identification of distinct subtypes and gene clusters, revealing profound prognostic differences between them (Figure 4C). A detailed examination revealed a significant variation in disulfidptosis score between the clusters, highlighting the precision of the method (Figure 4D, 4E). Additionally, disparities in DRG portrayals across disulfidptosis scores were observed (Figure 4F), emphasizing the discerning power of the method. Survival analysis within the training set revealed a marked prognostic divide, underlined by statistical significance (Figure 5A). The area under the curve (AUC) confirmed the efficacy of the prognostic model over various time frames (Figure 5B), while comparative survival analysis validated the superior prognosis of the low-risk group. Replicating these analyses in both the full cohort and the test set confirmed the model’s robust predictive ability (Figure 5C–5F), with ROC curves affirming its accuracy (Figure 5E, 5F). Along with age and sex differences, survival outcomes differed significantly between risk groups (Figure 5G). A comprehensive nomogram incorporating multiple clinical factors was used as an advanced prognostic tool (Figure 5K), with calibration curves demonstrating its accuracy (Figure 5L). Decision curve analysis confirmed the utility of the nomogram in predicting survival outcomes at various intervals, demonstrating its potential for improving BLCA patient prognosis (Figure 5H–5J).

### The disulfidptosis score and its association with tumor mutation burden and genomic instability

**Figure 6:**
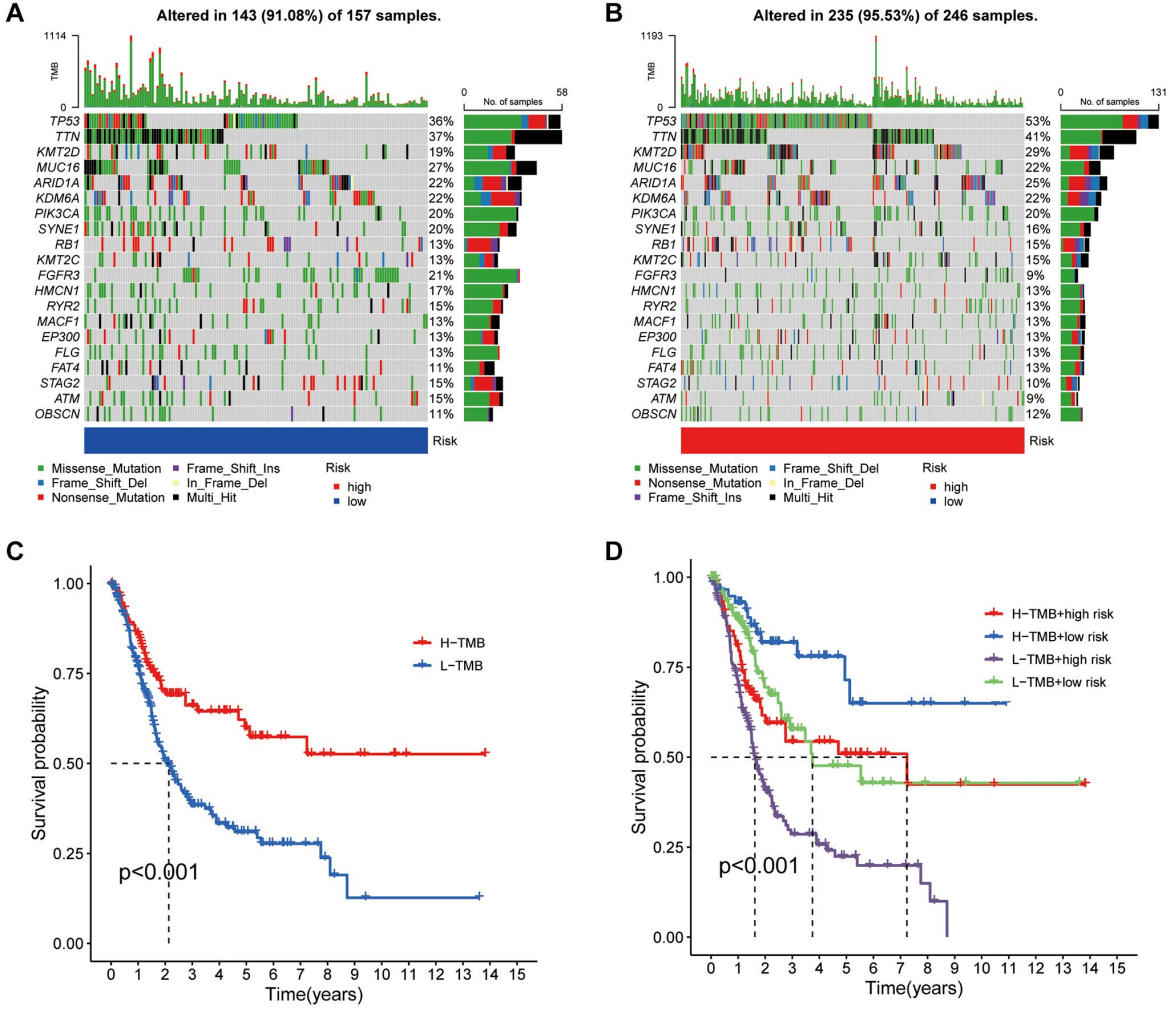
**Mutation analysis based on the risk score model.** (**A**, **B**) Waterfall plots summarizing the mutation status of high and low-risk patients. (**C**) Kaplan-Meier curves of high and low TMB groups. (**D**) Kaplan-Meier curves of four groups classified by risk score and TMB.

Genetic alterations play a pivotal role in cancer research. This investigation utilized somatic mutation data alongside disulfidptosis evaluations from TCGA to explore the implications of these data in BLCA. Our analysis highlighted TP53, TTN, and KMT2D as genes prevalently mutated in both risk groups (Figure 6A, 6B). The analysis also distinguished patients into groups with a tumor mutational burden (TMB) higher or lower than the median, revealing that those with a higher TMB exhibited improved survival outcomes, positioning TMB as a potential prognostic indicator (Figure 6C). Dividing the patient sample further into four quadrants based on TMB and risk scores clarified the impact of these variables on survival, with a low TMB and high-risk scores indicating the poorest outcomes (Figure 6D). This research thus contributes to our understanding of the genetic landscape and prognostic nuances of BLCA.

### Immune cell infiltration characteristics and biological behaviors between igh- and low-risk groups

**Figure 7:**
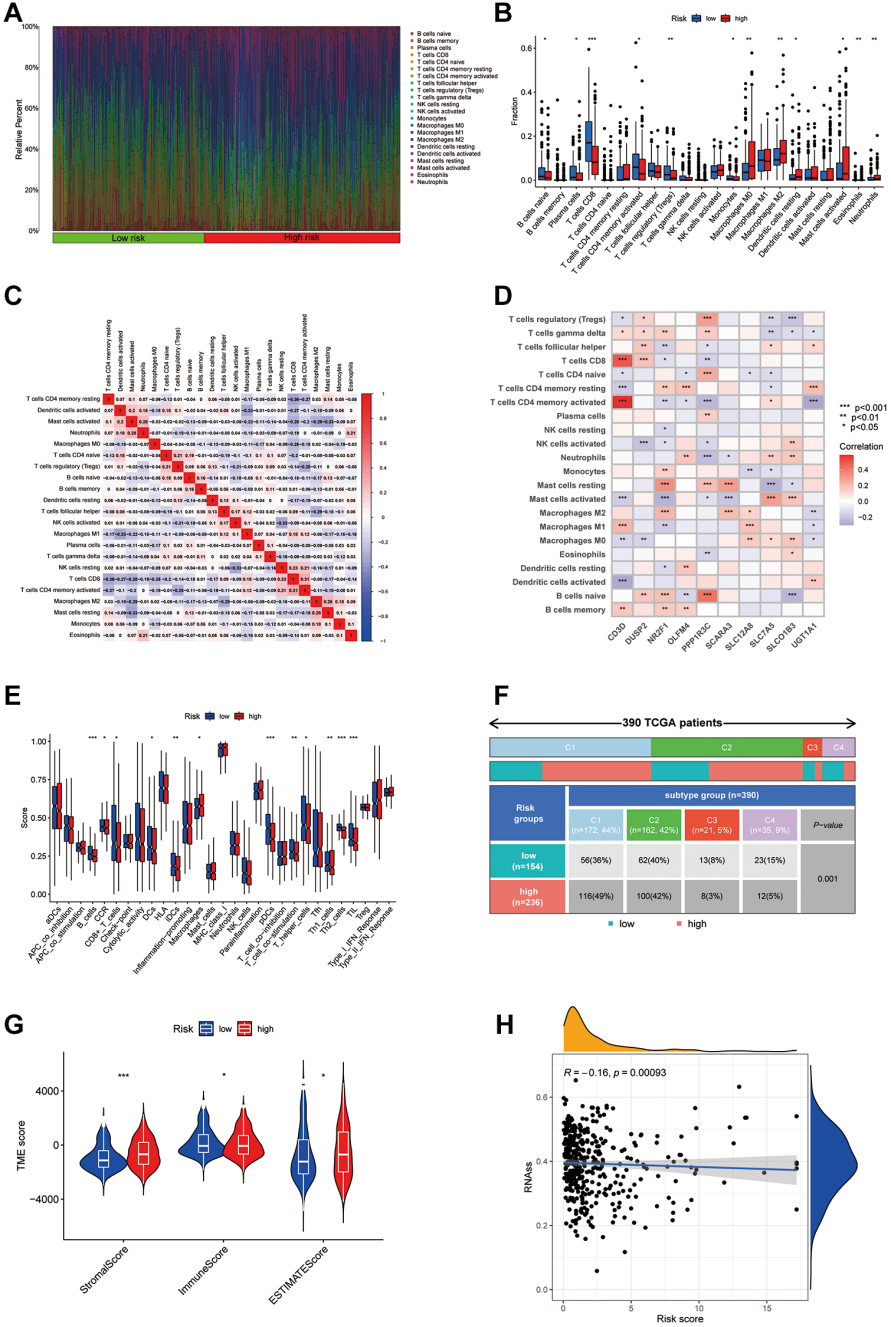
**Analysis of the immune microenvironment in different risk groups.** (**A**) Differences in immune infiltration status between different risk groups were evaluated by cibersort algorithms. (**B**) Differences in immune cell infiltration between different risk groups. (**C**) Correlation between the expression of immune cell. (**D**) Correlation between the expression of hub disulfidptosis risk score genes and immune cells. (**E**) Differences in ICI scores between different risk groups. (**F**) The immune subtype of patients in high- and low-risk groups. (**G**) Violin plot of stromal score, immune score, and estimate score between low and high-risk groups. (**H**) Correlation between the risk score and RNAss.

Using the CIBERSORT algorithm, 22 types of infiltrating immune cells were quantified in both cohorts (Figure 7A, 7B). Figure 7C illustrates the complex interactions among these immune cells. Notably, there was a significant variation in the presence of immune cells between the groups, with CD8+ T cells and Tregs showing reduced levels in the high-risk group, whereas M0 and M2 macrophages were elevated (Figure 7A). We also explored correlations between disulfidptosis-associated genes (PPP1R3C, SLC12A8, etc.) and immune cells using Pearson correlation analysis, as shown in Figure 7D. Further analysis using the ssGSEA method highlighted distinct immune landscape differences between the risk groups, particularly in B cells and Th2 cells (Figure 7E), and underscored a significant divergence in risk scores within immune subtypes (Figure 7F). Violin plots provided a visual comparison of stromal and immune scores and tumor purity between the risk groups, indicating notable differences (Figure 7G). Additionally, an inverse relationship was found between tumor stemness (RNA levels) and risk score, suggesting a decrease in stemness in the high-risk group (Figure 7H).

### The disulfidptosis score can predict immunotherapy efficacy

**Figure 8:**
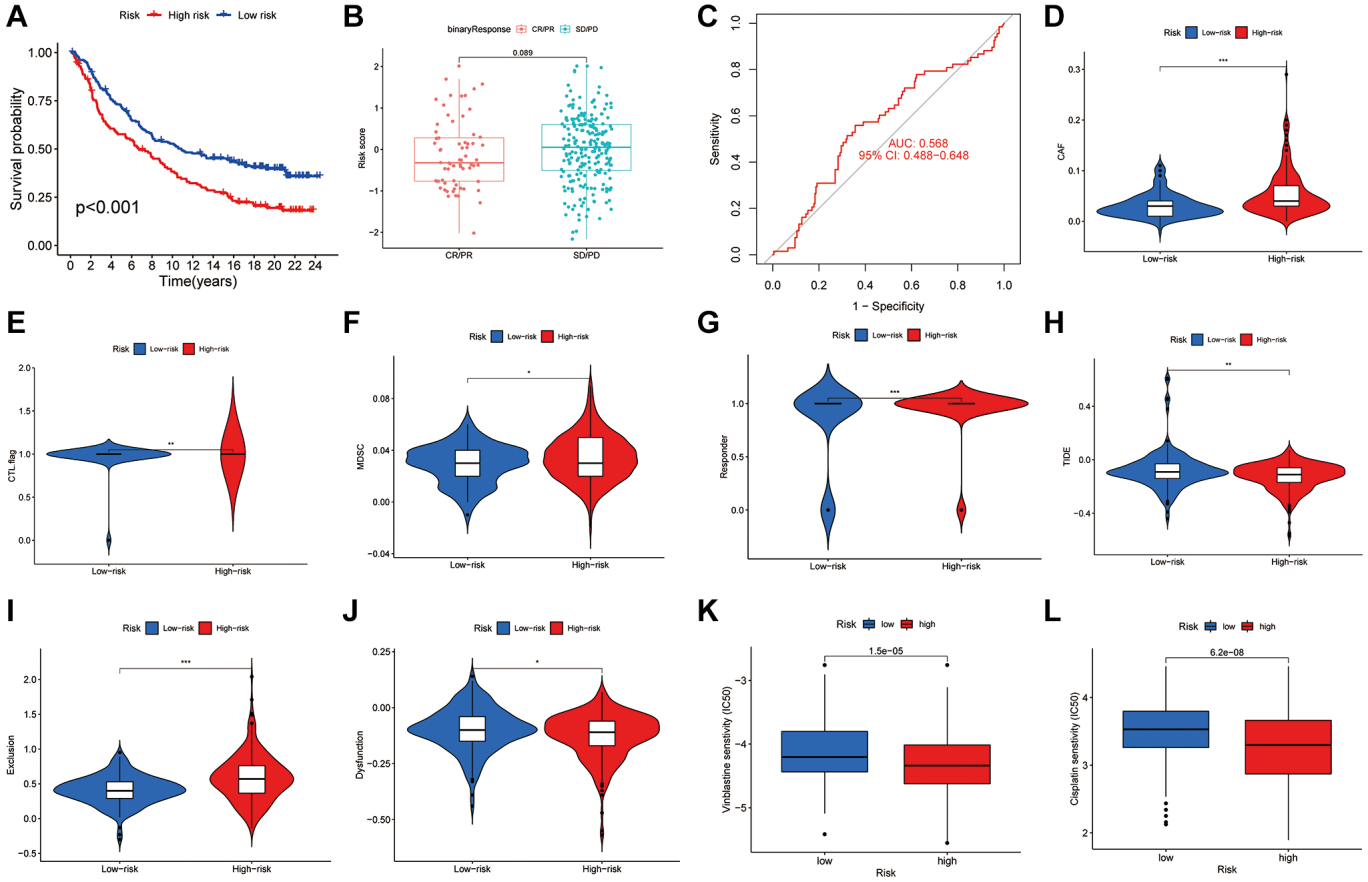
**The role of disulfidptosis patterns in immunotherapy and chemotherapy.** (**A**) Kaplan-Meier curves of high and low-risk groups in IMvigor210. (**B**) The difference of disulfidptosis score between treatment outcome groups. (**C**) ROC curves of the nomogram score system for BLCA patients in IMvigor210. (**D**–**J**) Correlation between risk score and Tide score. (**K**–**L**) Correlation between risk score and Vinblastine and cisplatin sensitivity.

The IMvigor210 cohort was analyzed to evaluate responses to anti-PD-L1 therapy, revealing a significant variation in risk scores among different response categories. Notably, a greater proportion of complete and partial responses was observed in the LR group (Figure 8A, 8B), with an AUC of 0.568 indicating the predictive value of the risk score (Figure 8C). Further analysis utilizing the TIDE score to predict immune evasion showed that individuals with lower risk scores were more likely candidates for immunotherapy (Figure 8D–8J). Additionally, we assessed the sensitivity of low-risk versus high-risk patients to the common BLCA chemotherapy agent vinblastine and cisplatin and revealed increased sensitivity in the low-risk group (Figure 8K, 8L). The disulfidptosis score thus emerges as a crucial metric for predicting immunotherapy outcomes and chemotherapy sensitivity in BLCA patients, underscoring its value in tailoring patient-specific treatment strategies.

### scRNA-seq analysis of ten hub disulfidptosis score-related genes in BLCA

**Figure 9:**
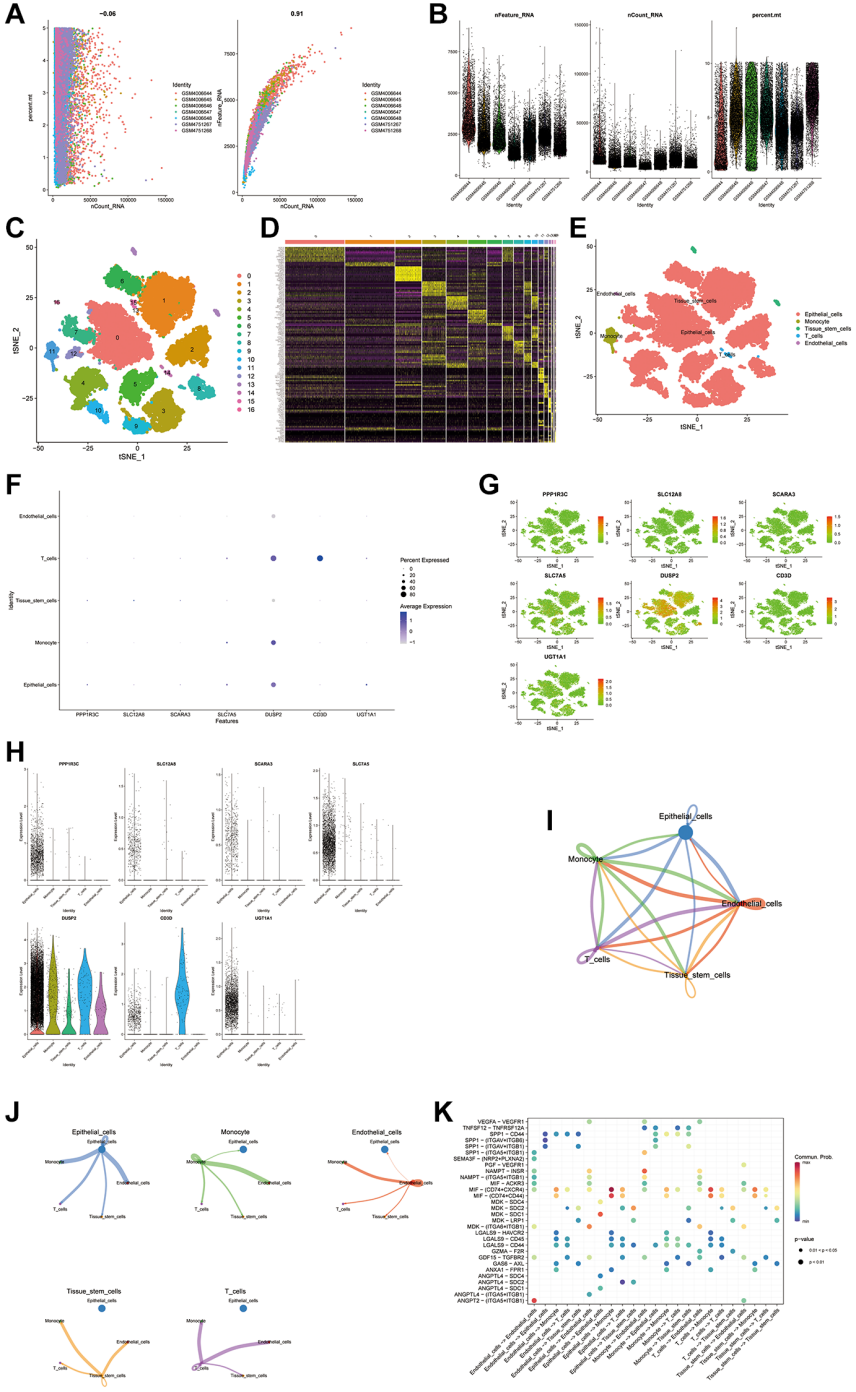
**scRNA seq analysis.** (**A**, **B**) Performing gene filtering, normalization, principal component analysis of scRNA seq data. (**C**–**E**) Annotation of all cell types in GSE135337 and the percentage of each cell type. (**F**–**H**) Expression of the genes in each cell type. (**I**–**K**) Cell to cell communications between each cell type.

Using the GSE135337 BLCA single-cell dataset, this study examined expression of ten pivotal genes associated with the disulfidptosis score within the TME. The initial steps included gene selection, data normalization, and dimensional reduction through principal component analysis, setting the resolution at 0.7 to delineate 37 distinct cell clusters, which was subsequently illustrated via t-SNE visualizations (Figure 9A–9C). Differential expression analysis highlighted cell type-specific genes (Figure 9D), with clusters annotated into five categories using marker genes from the CellMarker database (Figure 9E). Examination of the ten genes at the single-cell level revealed their expression across five cell types, with seven genes being actively expressed (Figure 9F–9H). Additionally, interactions within the TME were mapped, particularly focusing on macrophage interactions with other cells and quantifying the interaction strength (Figure 9I) while noting prevalent interactions with monocyte cells among all cell types (Figure 9J). A bubble plot was constructed to detail cell-to-cell communication among the five cell types, offering insights into their interplay (Figure 9K).

### DUSP2 and SLCO1B3 were differentially expressed in BLCA and had effects on malignant behaviors in BLCA cells

**Figure 10:**
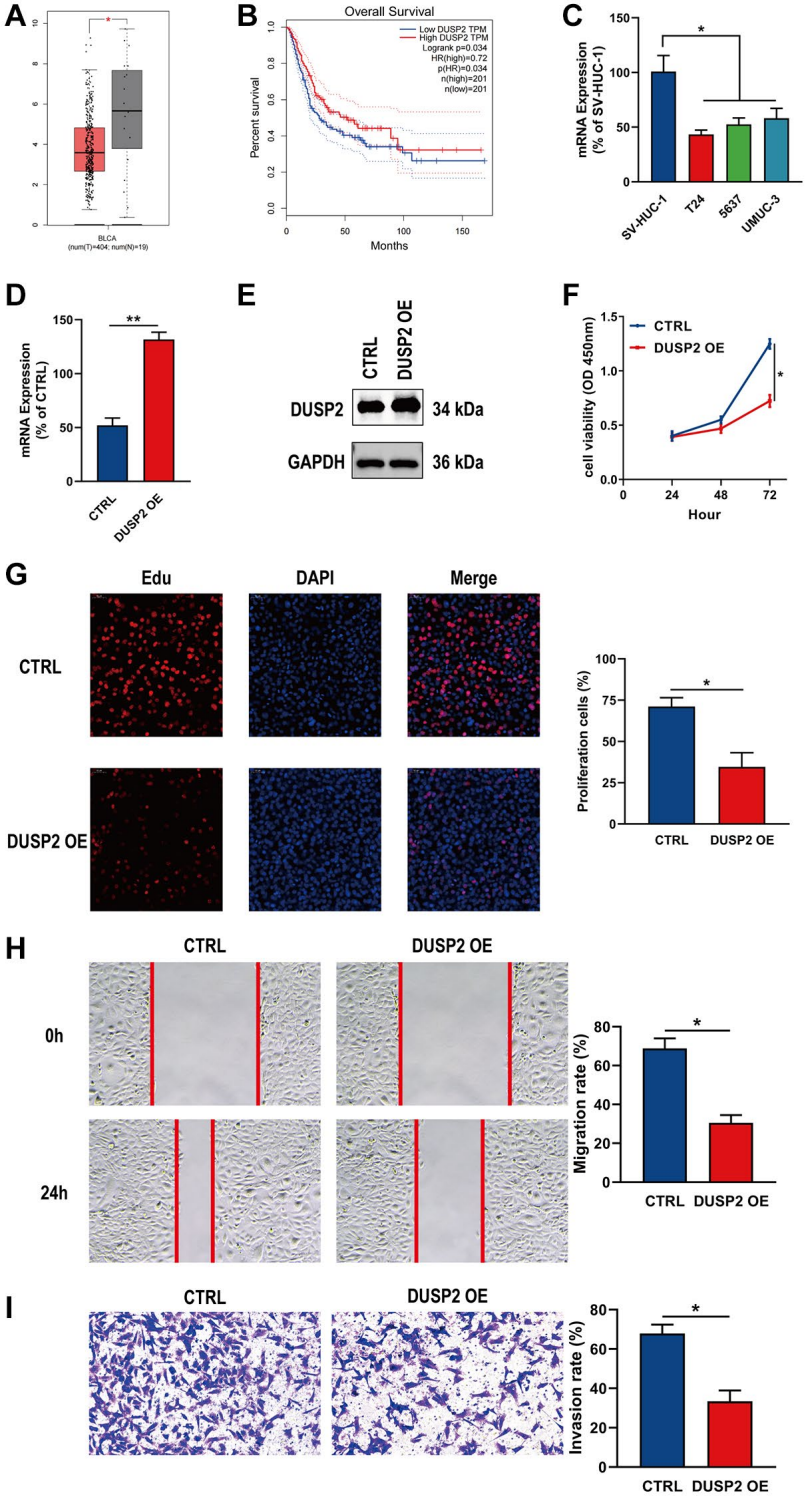
**Overexpression of DUSP2 inhibited the proliferation, migration, and invasion of BLCA cells.** (**A**, **B**) Differential analysis and survival analysis of DUSP2. (**C**) The mRNA expression of DUSP2 in BLCA cells and normal bladder cells. (**D**, **E**) The overexpression efficiency of DUSP2 was confirmed by qRT–PCR and western blotting. (**F**, **G**) CCK-8 and EdU assay were conducted to evaluate the proliferation ability. (**H**) Wound healing assay for the migration ability. (**I**) Transwell assay for the invasion ability. ^*^P < 0.05, ^**^P < 0.01.

Finally, we performed expression and survival analyses on the ten genes included in the prognostic signature. Among them, DUSP2 and SICO1B3 were selected as key genes. At the single-cell level, the expression of DUSP2 and SLCO1B3 was significantly different between tumor tissue and normal tissue. Notably, the difference was more significant between bladder tumor epithelial cells and normal tissue (Supplementary Figure 1). Through differential expression analysis, we found that expression of DUSP2 was markedly lower in BLCA tissues than in normal tissues (Figure 10A). Kaplan-Meier survival analysis further demonstrated that DUSP2 was associated with worse OS in BLCA patients, suggesting that DUSP2 may be a prognostic indicator of BLCA (Figure 10B). DUSP2 mRNA expression was much lower in BLCA cells than in normal bladder cells (Figure 10C). Finally, to further investigate the biological role of DUSP2, functional experiments were conducted. The transfection efficiency of DUSP2 was validated by qRT-PCR and western blotting (Figure 10D, 10E). CCK-8, EdU, wound healing, and Transwell invasion assays indicated that overexpressing DUSP2 significantly inhibited the proliferation, migration, and invasion of BLCA cells, suggesting that DUSP2 may play a tumor-suppressive role in BLCA (Figure 10F–10I).

**Figure 11:**
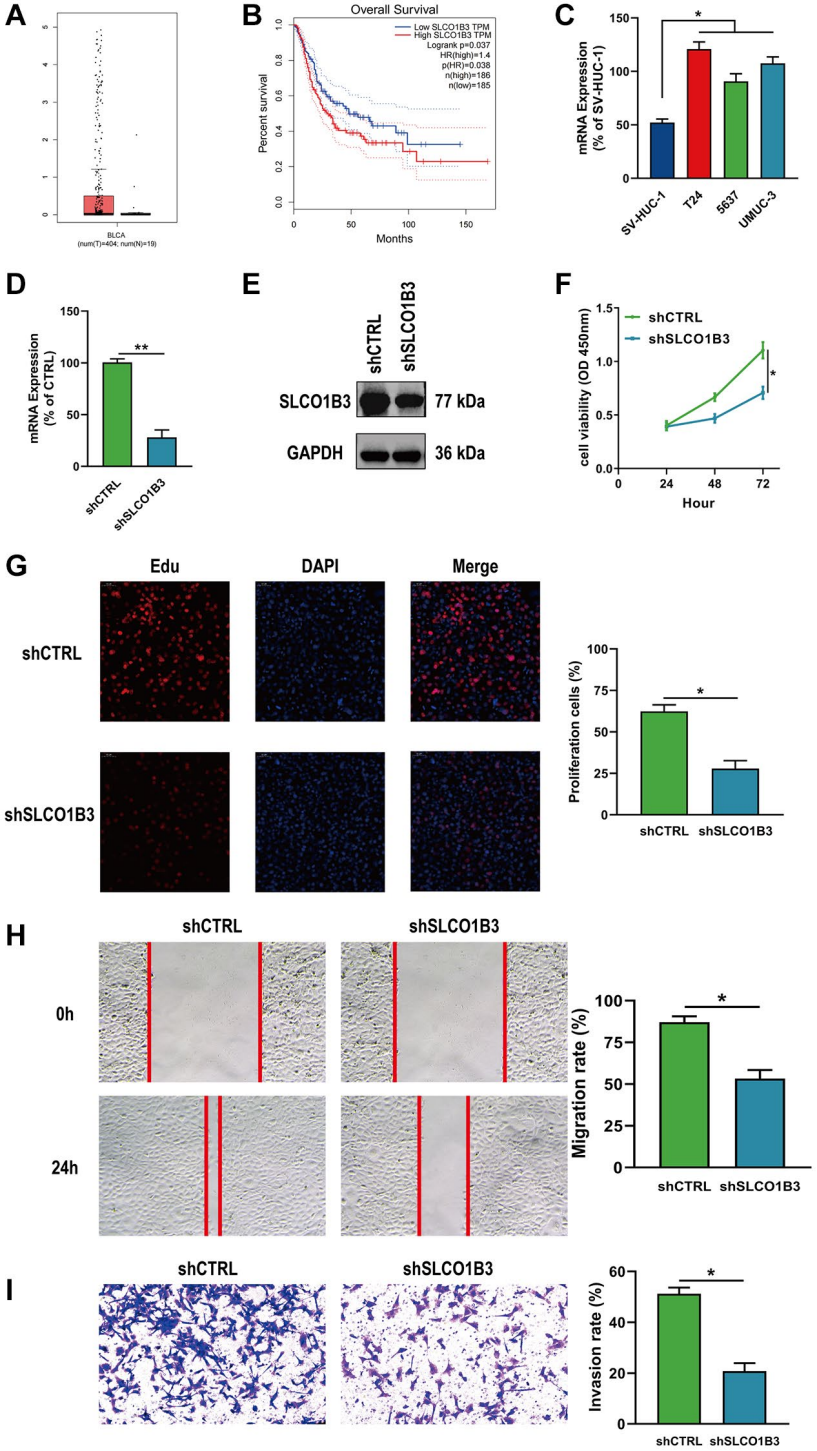
**Downregulation of SLCO1B3 inhibited the proliferation, migration, and invasion of BLCA cells.** (**A**, **B**) Differential analysis and survival analysis of SLCO1B3. (**C**) The mRNA expression of SLCO1B3 in BLCA cells and normal bladder cells. (**D**, **E**) The knockdown efficiency of SLCO1B3 was confirmed by qRT–PCR and western blotting. (**F**, **G**) CCK-8 and EdU assay for the proliferation ability. (**H**) Wound healing assay for the migration ability. (**I**) Transwell assay for the invasion ability. ^*^P < 0.05, ^**^P < 0.01.

Differential analysis revealed that SLCO1B3 was significantly upregulated in BLCA tissues compared to normal tissues (Figure 11A). Kaplan-Meier survival analysis revealed that SICO1B3 was associated with poor prognosis in BLCA patients, demonstrating the potential prognostic role of SLCO1B3 (Figure 11B). mRNA expression of SLCO1B3 was significantly greater in BLCA cells than in normal bladder cells (Figure 11C). Expression of SLCO1B3 was downregulated in T24 cells. As in the DUSP2 experiments described above, functional assays were performed, and the transfection efficiency was validated by qRT-PCR and western blotting (Figure 11D, 11E). CCK-8, EdU, wound healing, and Transwell invasion assays demonstrated that knockdown of SICO1B3 significantly suppressed the proliferation, migration, and invasion of BLCA cells, suggesting that SICO1B3 acts as a tumor-promoting gene (Figure 11F–11I).

## DISCUSSION

BLCA ranks as a major urological cancer worldwide [27]. Treatment paradigms for BLCA have evolved, highlighting the effectiveness of cisplatin-based chemotherapies and the potential of immunotherapy with immune checkpoint inhibitors (ICIs), despite variable patient responses [28]. The concept of disulfidptosis is emerging as a promising area for innovative treatments, focusing on the tumor’s redox state and disulfide metabolism [29–30]. Research indicates that targeting disulfidptosis could offer new therapeutic avenues, as some tumors adapt their redox balance for survival and interact with treatments such as paclitaxel [31]. The biological role of disulfidptosis in lung and colon adenocarcinoma has been investigated [32, 33]. Nonetheless, there is a lack of researches in BLCA.

In this investigation, we scrutinized genetic disparities within BLCA focusing on DRGs. MYH10 emerged as the most commonly mutated gene. A significant occurrence of CNVs in DRGs was observed, with a balanced distribution between deletions and amplifications, highlighting specific genes such as SLC3A2 and ACTN4 for amplification and FLNB for deletion. This analysis explored how these genetic changes influence mRNA expression, suggesting that CNVs may influence DRG expression levels. Amplification in certain DRGs corresponded with upregulation in cancerous tissues, in contrast to downregulation in other tissues. This study further delineated two disulfidptosis phenotypes, A and B, with distinct prognostic implications and immune cell infiltration patterns in the TME, underscoring the critical role of the TME in disulfidptosis and potential therapeutic targets for BLCA [34]. Through unsupervised clustering, two distinct disulfidptosis-associated gene clusters were discerned, with Cluster A linked to enhanced survival outcomes compared to the less favorable prognosis of Cluster B. Additionally, the study highlighted the TMB as a pivotal factor in cancer progression and patient survival, underscoring its potential as a prognostic marker in BLCA. These findings align with existing research, reinforcing the prognostic value of the TMB in the context of BLCA. This insight underscores the importance of genetic profiling in predicting patient outcomes and tailoring therapeutic strategies [35].

Most importantly, to deeply investigate the characteristics of disulfidptosis in BLCA, a disulfidptosis prognostic risk model was built, and its accuracy was verified. The model effectively predicted patient prognosis, immune infiltration, and drug efficacy. Compared with the disulfidptosis risk model of BLCA published previously [14], our model had a greater ability to predict prognosis and immune infiltration. Previous research has shown that a greater degree of infiltration of CD8+ T cells may indicate better prognosis and immunotherapy response [36]. This is consistent with the present results. We investigated differences in the proportions of infiltrating immune cells between two groups, with the low-risk group showing an increase in CD8+ T cells. These patients had better prognosis and immunotherapy responses than did those in the high-risk group. However, our results contradicted the findings for disulfidptosis in liver cancer, indicating that increased CD8+ T cells lead to worse prognosis [37]. This can be explained as follows: infiltration of CD8+ T cells may trigger mutations in cancer cells, thereby enhancing their immune escape ability. The expression levels of some genes were utilized to develop a risk score, which effectively differentiated disulfidptosis Clusters A and B, as well as their gene Clusters A and B. The disulfidptosis score showed good prediction performance across multiple cohorts, and this finding was validated in test cohorts.

Then, we identified two hub genes in the prognostic signature. Among the ten genes, DUSP2 and SLCO1B3 were found to be potential therapeutic targets because they were differentially expressed in BLCA and their expression levels were associated with patient prognosis. DUSP2 is a member of the nuclear type I DUSP family that may activate MAPKs, thereby preventing tumor progression [38]. Studies have demonstrated that DUSP2 is an important kinase in tumors. Moreover, it was reported that DUSP2 is downregulated in BLCA tissues, which is associated with poor prognosis in BLCA patients [39]. However, the biological role of DUSP2 in BLCA remains to be characterized. Through *in vitro* assays, this study explored the expression and biological role of DUSP2 in BLCA for the first time. DUSP2 was downregulated in BLCA cells and acted as a tumor-suppressor gene. It inhibited the proliferation, migration, and invasion of BLCA cells. Solute carrier organic anion transporter family member 1B3 (SLCO1B3) is a membrane-bound multispecific transporter found in hepatocytes. It transports several endogenous and exogenous compounds. SLCO1B3 contributes to the development of several cancers and regulates tumor sensitivity to chemotherapy [40, 41]. Nonetheless, its expression and biological function in BLCA have never been reported. Our results indicate that SLCO1B3 was upregulated in BLCA cells and acted as an oncogene. It also enhanced the proliferation, migration, and invasion of BLCA cells.

Although our prognostic signature related to disulfidptosis showed good performance, there are still some shortcomings. First, the use of data obtained from public databases might introduce biases. More prospective trials are needed to validate the findings in real-world studies. Additional *in vitro*/*in vivo* mechanistic experiments should also be conducted to expand our results. In the future, we will identify appropriate solutions to address these problems.

In summary, this study expands our understanding of DGRs and their role in BLCA. Moreover, we developed a prognostic tool comprising clinical parameters and risk assessments for predicting the prognosis and immunotherapy response of BLCA patients. We also identified two key DEGs and validated their expression and biological roles in BLCA, providing new targets for treating BLCA. The developed predictive signature may serve as a promising strategy for BLCA treatment.

**Figure 12:**
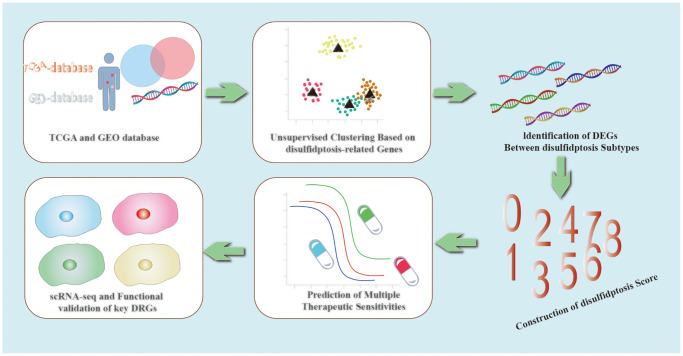
Graphical Abstract.

The Graphical Abstract is shown in Figure 12.

## Supplementary Materials

Supplementary Figure 1
