# Supplementary material for: Comprehensive analysis of scRNA-seq and bulk RNA-seq reveal the characteristics of disulfidptosis and a prognostic signature in BLCA
Source: Aging (Albany NY). 2024 Mar 20;16(6):5751–71. doi: 10.18632/aging.205686 (PMC11006495; doi:10.18632/aging.205686)
Supplement: Supplementary Figure 1 [file aging-16-205686-s001.pdf]

SUPPLEMENTARY FIGURE

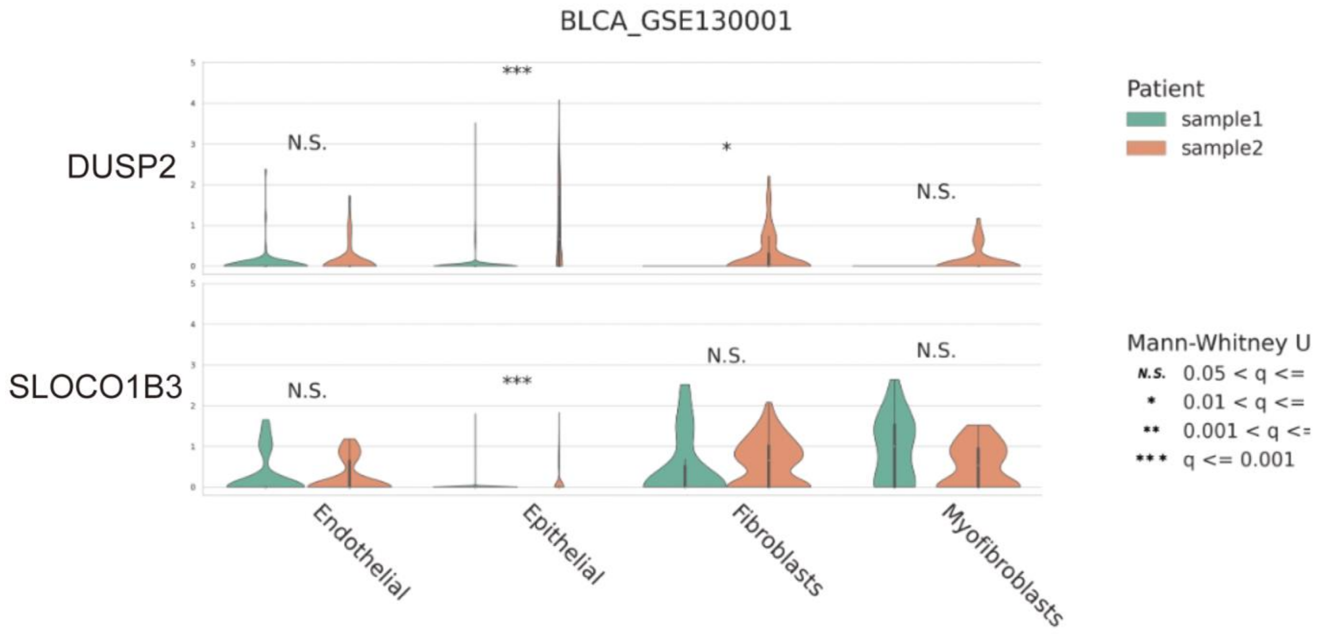

Supplementary Figure 1. Single-cell RNA sequencing analysis of DUSP2 and SCL1BO3. The analysis was performed on TISCH database. $^{*}P < 0.05$, $^{**}P < 0.01$.
